# Artificially decreased dissolved oxygen increases the persistence of *Trichomonas gallinae* in water

**DOI:** 10.1016/j.ijppaw.2019.04.002

**Published:** 2019-04-07

**Authors:** Kathryn Purple, Todd Amacker, Chauntelle Williams, Richard Gerhold

**Affiliations:** a Comparative and Experimental Medicine, University of Tennessee College of Veterinary Medicine, 2407 River Drive, Knoxville, TN, 37996, USA; b Department of Biomedical and Diagnostic Sciences, University of Tennessee College of Veterinary Medicine, 2407 River Drive, Knoxville, TN, 37996, USA; c Department of Forestry, Wildlife, and Fisheries, University of Tennessee Institute of Agriculture, 274 Ellington Plant Sciences Building, Knoxville, TN, 37996, USA

**Keywords:** *Trichomonas gallinae*, Persistence, Bird waterers, Dissolved oxygen, Avian diseases, Oxyrase^®^

## Abstract

Water containing organic material has been shown to increase the persistence of the avian pathogenic protozoa, *Trichomonas gallinae*. We hypothesized that the decrease in dissolved oxygen due to microbes in the organic material could increase persistence of the microaerophilic trichomonads. Using simulated birdbaths, we determined 1) the levels of dissolved oxygen in distilled water with various amounts of organic material, 2) the concentration of the oxygen-scavenging enzyme Oxyrase^®^ needed to achieve the dissolved oxygen levels obtained in organic material contaminated water, and finally, 3) the persistence of two *T. gallinae* isolates in Oxyrase®-supplemented water. An average of 9.6% dissolved oxygen was obtained with the addition of 15 g organic material to 500 ml of distilled water, whereas organic material-free water had 86.2% dissolved oxygen. The addition of 0.5% and 1.0% (vol/vol) Oxyrase^®^ to organic material-free water yielded dissolved oxygen of 18.6% and 6.9%, respectively. Using 0.5% and 1.0% concentrations of Oxyrase^®^, we evaluated the persistence of two trichomonad isolates by inoculating ∼1 million trichomonads into 500 ml distilled water in triplicate. At various time-points, 0.5 ml aliquots of trichomonad-inoculated water were obtained and placed into Hollander Fluid media, incubated at 37 °C, and read by light microscopy every other day for 5 days. In our 1% Oxyrase^®^ treatments, the longest recorded persistence of broad-winged hawk 1 increased from the previously reported 4hrs to 30hrs and Cooper's hawk 4 from 16hrs to 30hrs. These results indicate that the mechanism for organic material-mediated trichomonad persistence is associated with decreased dissolved oxygen, further demonstrating the importance of keeping birdbaths free of organic debris to discourage trichomonad persistence.

## Highlights

• Low (9.6%) dissolved oxygen (DO) levels were achieved in waterers with addition of organic material (OM).
• Addition of 1% Oxyrase resulted in similar DO findings (6.9%) with a pH of 7.24
• Lower DO is likely mechanism of increased *Trichomonas gallinae* persistence in waterers with OM.
• Results underscore the need to keep bird waterers clean to minimize *T. gallinae* transmission.

## Graphical abstract

**Figure:**
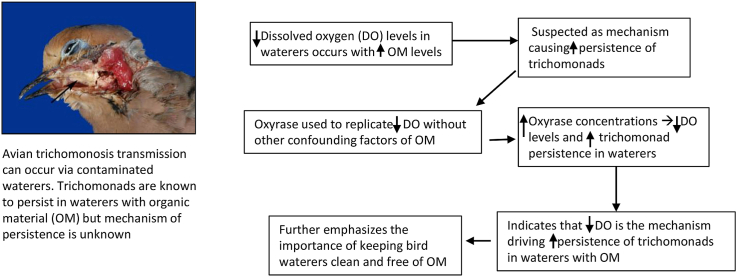

## 1 Introduction

The avian protozoan parasite *Trichomonas gallinae* has been, and continues to be, an important cause of mortality in many bird species (Amin et al., 2014; Stabler, 1947) and is typically transmitted from rock pigeons (*Columba livia*) to other Columbiformes (Bunbury et al., 2007; Girard et al., 2014), raptors (Boal et al., 1998; Martínez-Díaz et al., 2015), and passerines (Forzán et al., 2010; Neimanis et al., 2010; Robinson et al., 2010). Transmission of trichomonads from Columbids to passerine species has been linked to backyard birdbaths and other contaminated water sources (Anderson et al., 2009; Forrester and Foster, 2008; Ganas et al., 2014; Stabler, 1954). Trichomonads were traditionally believed to be labile in the environment due to the lack of a true cyst phase (Kocan, 1969); however, recent research has shown that trichomonads persist in various simulated environmental conditions in water (Gerhold et al., 2013; Purple and Gerhold, 2015; Purple et al., 2015) and on moist bird seed (McBurney et al., 2017). The addition of organic material (OM) including leaves, soil, and other detritus, has consistently resulted in increased persistence of *T. gallinae* in water (Gerhold et al., 2013; Purple and Gerhold, 2015; Purple et al., 2015), although the reason remains unknown. The added organic components may include a variety of environmental microorganisms. The relationships among *Trichomonas* spp., environmental bacteria, and other soil-dwelling protists added by OM are suspected to play a role in the increased persistence of trichomonads in distilled water.

*Trichomonas gallinae* contains hydrogenosomes, an evolutionary mitochondrial replacement organelle (Makiuchi and Nozaki, 2014), to thrive in the microaerophilic microclimate in the oral cavity created by the parasite's destruction of the esophageal lining and the resulting caseous lesions. We hypothesized that environmental microorganisms within OM may consume oxygen and create microaerophilic microclimates, thus providing a mechanism by which OM increases the persistence of *T. gallinae* in water.

To determine the relationship between dissolved oxygen (DO) and trichomonad persistence in the absence of the confounding properties of OM (i.e. provision of nutrients, alterations in pH, or other unkown factors) we recorded 1) the levels of DO in water due to the addition of various amounts of OM, 2) the concentration of the oxygen scavenging enzyme Oxyrase^®^ needed to achieve the DO levels in OM-contaminated water, and finally, 3) the persistence of two *T. gallinae* isolates in Oxyrase®-supplemented water containing DO concentrations found in OM-contaminated water. Oxyrase^®^ is a commercially available enzyme system that decreases DO in water (Oxyrase^®^, Inc., Mansfield, OH). It is effective over a wide temperature range (5–65 °C) and a wide pH range (6.8–9.4) (Oyrase^®^ white paper).

## 2 Materials and methods

### 2.1 Measuring DO levels with added OM

Organic material, including deciduous leaf litter, soil, and other detritus, was collected from the same local natural area as discussed in previous persistence studies (Purple and Gerhold, 2015). Organic material in amounts of 1, 5, 10, and 15 g (with 0 g as a negative control) was added to 500 mL of distilled water in plastic containers in 3 replicates. Dissolved oxygen and temperature were measured with a Sper Scientific DO Meter (Scottsdale, AZ) at 5 time points after the OM was added to the water (0min, 30min, 24hr, 48hr, 72hr). pH was measured at the 24hr, 48hr, and 72hr time points with a Denver Instrument UltraBASIC pH/mV meter (Arvada, CO). Both meters were calibrated per manufacturer's instructions before each time point.

### 2.2 Measuring DO with added Oxyrase^®^

To establish the amount of Oxyrase^®^ needed to decrease DO to levels achieved with the addition of OM found in the preceding experiment, we added 2 amounts of Oxyrase^®^ (2.5 mL and 5 mL) to 500 mL of distilled water (0.5% and 1% vol/vol, respectively). Dissolved oxygen, temperature, and pH were measured as above at the 24hr, 48hr, and 72hr time points.

### 2.3 Measuring persistence of trichomonads in water with decreased DO

Using the above results, we re-created the levels of DO found in OM-supplemented distilled water by adding 0.5% and 1% Oxyrase^®^ (vol/vol) to plastic containers with 500 mL of distilled water. We evaluated two trichomonad isolates: one from Cooper's hawk 4 (COHA) and one from broad-winged hawk 1 (BWHA), which were used in previous persistence trials (Gerhold et al., 2013; Purple and Gerhold, 2015; Purple et al., 2015). Both of these hawks showed pathologic lesions consistent with trichomonosis characterized by fulminate oral necrosis. *Trichomonas* was collected at necropsy, cultured in Diamond's media (Diamond, 1957) with antibiotics until cultures were axenic, and cryopreserved in liquid Nitrogen. These isolates were analyzed with PCR to amplify the ITS region and these sequences were compared with other *Trichomonas* isolates (Gerhold et al., 2008). The COHA sequence was in ITS group A and the BWHA in group B.

Before *Trichomonas* inoculation in our simulated birdbaths, isolates were revived from cryopreservation in Hollander media (Smith, 1983) with supplemental fetal bovine serum, antibiotics, and antifungals as previously reported (Purple and Gerhold, 2015). After logarithmic growth was achieved, trichomonads were counted using a hemocytometer, and cultures were adjusted to a concentration of 10^6^ trichomonads/mL of media. The inoculating dose of 10^6^ trichomonads was used to allow comparison with live animal infection studies using 10-50^5^ (Kocan and Knisley, 1970), 20^4^ organisms to test *in vitro* drug efficacy (Franssen and Lumeij, 1992), and 10^6^ as in our previous persistence work.

For the persistence experiment, containers were filled with 500 mL of bottled, distilled water. Oxyrase^®^ was pipetted into the water in the amounts of 0.5 and 2.5 mL. At time point 0 min, 1 mL of each trichomonad culture was added to individual simulated birdbaths. The water was immediately stirred with the pipette tip and the 0 min sample was taken. Samples (aliquots of 0.5 mL from the baths taken at 4, 8, 13, 18, 26, and 30hrs) were introduced into the Falcon^®^ 25 mL plug-sealed tissue culture flasks (Corning Inc., Corning, NY) with 5 mL Hollander fluid media. Flasks were incubated at 37 °C for 5 days. On days 1, 3, and 5 post-inoculation, flasks were examined by light microscopy to detect the characteristic movement of live trichomonads. Positive flasks containing at least one live trichomonad were recorded, and negative flasks were read until day 5. Research and the personal experience of the authors reveal that samples still negative at day 5 are unlikely to become positive (Bunbury et al., 2005; Cover et al., 1994). Dissolved oxygen and temperature were measured as above at 0hr (after Oxyrase^®^, but before trichomonads were added), 8hr, and 30hr time points. pH was measured at hour 0 and 30. The accession number for the COHA-4 is EU215369, and the number for the BWHA-1 is EU215368.

## 3 Results

### 3.1 Measuring DO levels with added OM

**Fig. 1:**
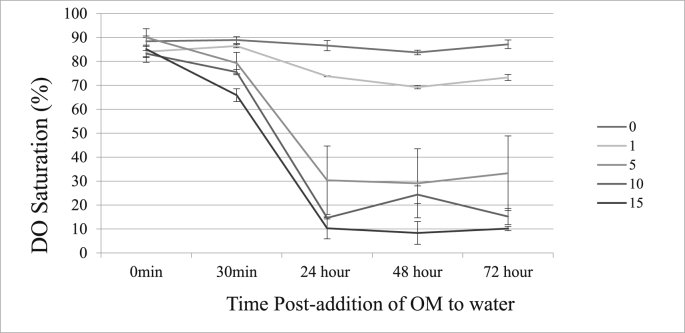
Dissolved oxygen (DO) saturation over time after the addition of different amounts of organic material (OM) to 500 mL distilled water in plastic containers. Error bars = standard deviation from 3 replicates. Legend title: OM (in grams).

The DO saturation with 15 g OM at 24hrs was 10.3% ( ±4.43) (Table 1). The DO in the control containers (0 g OM) at 24 h was 86.6% ( ±2.12). Dissolved oxygen decreased proportionally as the amount of OM increased in the simulated birdbaths (Fig. 1). At all time points (0.5, 24, 48, 72hrs) excluding time 0hr, DO was highest in the control container and was lower in the remaining treatments. Containers with OM had an average pH of 7.0 ( ±0.07) at 24 h (Table 1), similar to 7.02, the pH in trichomonad-specific growth media. Containers without OM had a pH of 5.84 at 24 h.

**Table 1:** Dissolved oxygen (DO) saturation and pH (with standard deviation) over time after the addition of different amounts of organic material (OM) in 500 mL distilled water in plastic containers.

Organic Material (g)	Hours post-addition of OM							
	0	0.5	24		48		72	
	DO (mean±SD)	DO (mean±SD)	DO (mean±SD)	pH (mean±SD)	DO (mean±SD)	pH (mean±SD)	DO (mean±SD)	pH (mean±SD)
0	88.42 ± 1.57	88.94 ± 1.42	86.6 ± 2.12	5.84 ± 0.10	84.74 ± 0.97	5.68 ± 0.0.22	87.14 ± 1.79	5.64 ± 0.27
1	84.05 ± 2.34	86.44 ± 0.73	73.83 ± 0.23	6.97 ± 0.08	69.27 ± 0.72	7.11 ± 0.11	73.32 ± 1.24	7.23 ± 0.14
5	90.0 ± 3.68	79.32 ± 4.45	30.37 ± 14.29	6.98 ± 0.07	29.09 ± 14.40	7.19 ± 0.05	33.29 ± 15.61	7.28 ± 0.07
10	83.3 ± 1.31	75.53 ± 1.08	14.65 ± 0.44	7.02 ± 0.03	24.37 ± 3.65	7.20 ± 0.06	15.22 ± 3.35	7.25 ± 0.02
15	85.1 ± 5.48	65.09 ± 2.68	10.31 ± 4.43	6.88 ± 0.07	8.35 ± 4.76	7.12 ± 0.16	10.19 ± 0.88	7.10 ± 0.08

Note from author: Table needs landscape view to avoid inappropriate line breaks. See separate Table 1 file.

### 3.1 Measuring DO with added Oxyrase^®^

Oxyrase^®^ added in 0.5% and 1.0% vol/vol resulted in DO saturations that averaged 18.7% and 6.9%, respectively, between the DO saturation range that results from the addition of 15 g OM, the previous amount used to measure persistence (Table 2). The 0% Oxyrase^®^ control container averaged 92% DO, similar to the 85.6% average for the 0 g OM control in the preceding experiment. The 0% Oxyrase^®^ control container had a pH of 5.1 averaged from time points 24, 48, and 72hrs, whereas the 0.5% and 1.0% treatments had average pHs of 7.13 and 6.88 over the same time points (Table 2).

**Table 2:** Dissolved oxygen (DO) saturation and pH (with standard deviation) over time after the addition of different percentage (% vol/vol) Oxyrase ^®^ to 500 mL distilled water in plastic containers.

Oxyrase ® (vol/vol)	Hours post-addition of Oxyrase ^®^					
	24		48		72	
	DO	pH	DO	pH	DO	pH
0%	89.50 ± 0.57	5.07 ± 0.25	92.59 ± 0.23	5.05 ± 0.20	94.02 ± 0.21	5.18 ± 0.31
0.50%	17.52 ± 3.32	6.89 ± 0.01	13.37 ± 12.19	6.99 ± 0.02	25.15 ± 9.26	7.52 ± 0.04
1%	6.00 ± 0.97	7.10 ± 0.02	5.30 ± 0.80	6.68 ± 0.21	9.35 ± 4.29	6.85 ± 0.08

### 3.3 Measuring persistence of trichomonads in water with decreased DO

**Fig. 2:**
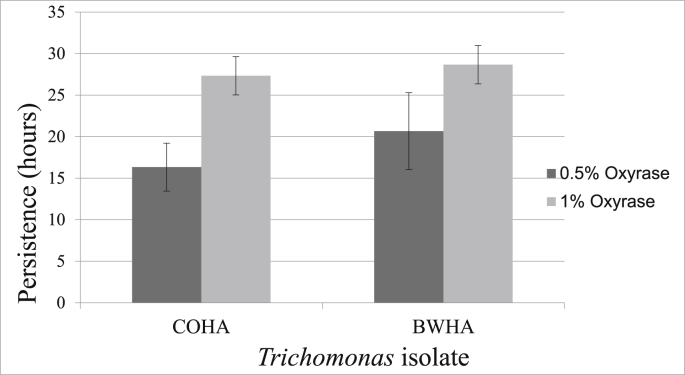
Persistence of two *Trichomonas* isolates (COHA, BWHA) in 500 mL distilled water in plastic containers with different concentrations (vol/vol) of Oxyrase^®^. Error bars = standard deviation from 3 replicates. Legend title: Concentration of Oxyrase^®^ (vol/vol).

Persistence in containers with 0.5% and 1% Oxyrase^®^ was greater than previously reported persistence in water with OM for both the COHA and BWHA isolates (Fig. 2). The experimental 0.5% and 1% Oxyrase^®^ treatments had 43.9% and 16.1% DO, respectively. In the present study, the maximum persistence occurred in the 1% Oxyrase^®^ treatments, where both the COHA and BWHA persisted to our final sampling time point of 30hrs. At time point 0hr, the 0.5% and 1.0% Oxyrase^®^ had an average pH of 7.13 and 7.24 for the COHA and 7.10 and 7.24 for the BWHA, respectively.

## 4 Discussion

We determined that artificially decreased DO using Oxyrase^®^ increased the maximum trichomonad persistence to 30hrs for both COHA and BWHA isolates. This increase is substantial compared to previous published studies using 15 gm of OM in which COHA isolate persisted 16hrs and BWHA isolate persisted 4hrs. We report maximum persistence because both isolates persisted until our final sampling time point (30hrs) in at least 1 replicate (1/3 for the COHA; 2/3 for the BWHA). Maximum persistence indicates the isolates persisted to the final sampling time point; however, the isolates may have remained viable even longer. Future persistence trials should extend past 30hrs to determine the potential end points.

Amoeba-bacteria and protozoan-bacteria relationships provide examples of a microorganism benefiting from the reduction in DO created by another. *Acanthamoeba castellanii,* a common environmental protist, has been shown to increase the survival of the microaerophilic bacterium and medically important pathogen, *Campylobacter jejuni,* by decreasing DO in a shared liquid medium (Bui et al., 2012). The same authors found a similar trend with the addition of the protozoa *Tetrahymena pyriformis,* another oxygen consumer, suggesting this phenomenon is not unique to *A. castellanii*.

We conclude that a reduction in DO as a result of adding OM is an important factor that leads to increased persistence of trichomonad isolates. This information will be helpful to the public and to biologists that provide supplemental water (i.e. birdbaths) for wildlife. Prompt removal of OM from these outdoor sources and mechanical aeration of the water could benefit birds that visit these places by increasing DO and making artificial waterers less suitable for *Trichomonas* spp. In particular, the removal or limiting of birdseed into waterers may be particularly important given previous findings of birdfeeders as a source of *T. gallinae* due to increased density of birds at feeders and contamination of feeders by the parasite (McBurney et al, 2015, 2017; Lawson et al., 2018).

Chemical treatment of water for bacterial or protist contamination is precluded by an inability to calculate and create disinfectant levels safe for bird and wildlife consumption in a volume that is ever-changing (due to refilling, rain, splashing, and evaporation). Aerators, like those used in fish tanks, would add DO to the water and could be explored as an option, although issues with power and function in the outdoors would have to be investigated.
