# Supplementary material for: Artificially decreased dissolved oxygen increases the persistence of Trichomonas gallinae in water
Source: Int J Parasitol Parasites Wildl. 2019 Apr 7;9:100–3. doi: 10.1016/j.ijppaw.2019.04.002 (PMC6463543; doi:10.1016/j.ijppaw.2019.04.002)
Supplement: Multimedia component 1 [file mmc1.docx]

**TABLE S1** Persistence of two trichomonad isolates with two different concentrations of Oxyrase® in 3 replicates with the recorded dissolved oxygen (DO) saturation for each time point. pH was also recorded at hour 30 and is shown with standard deviation. COHA= Cooper’s hawk; BWHA= broad-winged hawk; STDEV= standard deviation

|  | | Oxyrase ®  (vol/vol) | |
| --- | --- | --- | --- |
|  |  | 0.5% | 1.0% |
|  | Replicate | Minimum persistence  (hours) | Minimum persistence  (hours) |
| COHA | 1 | 13 | 26 |
|  | 2 | 18 | 26 |
|  | 3 | 18 | 30 |
|  | Mean +/- SD | 16.33 +/- 2.89 | 27.33 +/- 2.31 |
| BWHA | 1 | 18 | 26 |
|  | 2 | 18 | 30 |
|  | 3 | 26 | 30 |
|  | Mean +/- SD | 20.67 +/- 4.62 | 28.67 +/- 2.31 |
